# An exploratory qualitative study of the prevention of road traffic collisions and neurotrauma in India: perspectives from key informants in an Indian industrial city (Visakhapatnam)

**DOI:** 10.1186/s12889-021-10686-z

**Published:** 2021-03-30

**Authors:** Santhani M Selveindran, Gurusinghe D. N. Samarutilake, K. Madhu Narayana Rao, Jogi V. Pattisapu, Christine Hill, Angelos G. Kolias, Rajesh Pathi, Peter J. A. Hutchinson, M. V. Vijaya Sekhar

**Affiliations:** 1 Department of Clinical Neurosciences, Addenbrooke’s Hospital, Cambridge University Hospitals Trust, Cambridge, UK; 2 NIHR Global Health Research Group on Neurotrauma, University of Cambridge, Cambridge, UK; 3 Ministry of Health, Colombo, Sri Lanka; 4 Department of Neurosurgery, King George Hospital, Visakhapatnam, India; 5 Department of Pediatric Neurosurgery, University of Central Florida College of Medicine, Orlando, Florida USA; 6 Cambridge Institute of Public Health, University of Cambridge, Cambridge, UK

**Keywords:** Road traffic collisions prevention, Neurotrauma prevention, India, Key informant perspectives

## Abstract

**Background:**

Despite current preventative strategies, road traffic collisions (RTCs) and resultant neurotrauma remain a major problem in India. This study seeks to explore local perspectives in the context within which RTCs take place and identify potential suggestions for improving the current status.

**Methods:**

Ten semi-structured interviews were carried out with purposively selected key informants from the city of Visakhapatnam, Andhra Pradesh. Participants were from one of the following categories: commissioning stakeholders; service providers; community or local patient group/advocacy group representatives. Transcripts from these interviews were analysed qualitatively using the Framework Method.

**Results:**

Participants felt RTCs are a serious problem in India and a leading cause of neurotrauma. Major risk factors identified related to user behaviour such as speeding and not using personal safety equipment, and the user state, namely drink driving and underage driving. Other reported risk factors included poor infrastructure, moving obstacles on the road such as other vehicles, pedestrians and animals, overloaded vehicles and substandard safety equipment. Participants discussed how RTCs affect not only the health of the casualty, but are also a burden to the healthcare system, families, and the national economy. Although there are ongoing preventative strategies being carried out by both the government and the community, challenges to successful prevention emerged from the interviews which included resource deficiencies, inconsistent implementation, lack of appropriate action, poor governance, lack of knowledge and the mindset of the community and entities involved in prevention. Recommendations were given on how prevention of RTCs and neurotrauma might be improved, addressing the areas of education and awareness, research, the pre-hospital and trauma systems, enforcement and legislation, and road engineering, in addition to building collaborations and changing mindsets.

**Conclusions:**

RTCs remain a major problem in India and a significant cause of neurotrauma. Addressing the identified gaps and shortfalls in current approaches and reinforcing collective responsibility towards road safety would be the way forward in improving prevention and reducing the burden.

**Supplementary Information:**

The online version contains supplementary material available at 10.1186/s12889-021-10686-z.

## Background

Neurotrauma is an increasing global health problem and a major cause of morbidity and mortality [1, 2]. The current evidence-base indicates that a disproportionate burden of neurotrauma, which refers to any traumatic injury to the brain and/or spinal cord, occurs in low-and-middle-income countries (LMICs), the largest cause being due to road traffic collisions (RTCs) [2–5].

India is an LMIC with one of the highest numbers of RTCs and road traffic-related injuries and deaths in the world [6, 7]. Over the last two decades, India has witnessed rapid urbanisation and motorisation which has led to an increase in vehicle density where local statistics have shown that while road length has increased by 39%, the number of motor vehicles have increased by 158% [7, 8]. This situation is believed to contribute to increasing numbers of RTCs, injuries and deaths over time [8, 9]. A recent publication from the Ministry of Road Transport and Highways reported a total of 467, 044 RTCs in 2018 with 469, 418 injured and 151,417 killed [7].

The Indian government have recognised this as a problem and have initiated many strategies to prevent RTCs, particularly in the last few years as a response to the United Nations ‘Decade of Action for Road Safety’ [6, 10]. Aside from a multi-pronged strategy based on education, engineering and enforcement, the government is also encouraging increased research into road safety and injury prevention [7].

Although there have been studies from India carried out in this area, most tend to utilise quantitative methods with a focus on risk factors and disease burden [6, 11]. While this type of research is helpful in defining the problem, qualitative research is instrumental in prevention as it provides insight into the environments and behaviours which make RTCs and resulting neurotrauma more likely, and which influence the uptake and implementation of prevention programmes [6, 12, 13].

Qualitative research also allows the exploration of perspectives from multiple stakeholders, particularly those involved in different facets of prevention of RTCs and neurotrauma, in order to ascertain their understanding of the phenomenon and the contextual factors that influence it [14]. These individuals, termed ‘key informants’ are not only informed about the topic of research, but would also be able to provide knowledge about local circumstances, community needs and perspectives [15]. As a result, key informants are often sought-after to provide recommendations to improve existing programmes [15, 16].

This current study was developed as part of a mixed-methods project named the Global Prevention of NeuroTrauma-Road Traffic Collisions (GPONT-RTC). This multi-centre project involves three neurosurgical centres in India, Pakistan and Colombia respectively, where one of the aims is to illustrate the current context within which RTCs and neurotrauma occur, and describe contextual factors that would affect and influence the development and implementation of preventative programmes. This ongoing project which commenced in December 2018, is part of the work that is being undertaken by the NIHR Global Health Research Group for Neurotrauma, and is led by the first author (SMS) [17].

As a study within this wider research project, the objective of this study was to explore key informants’ views on and experiences of RTCs and related neurotrauma in their context, and on the current preventative strategies, including factors affecting the implementation of these strategies. We also sought to identify potential suggestions for improving the current state of RTCs and neurotrauma prevention. The research questions for this study can be found in Table 1.

**Table 1:** Study research questions

• What are key informants’ views and/or experiences of RTCs and RTC-related neurotrauma?	
• What are current approaches to preventing RTCs and related neurotrauma?	
• What affects the successful implementation of these approaches?	
• What is the best practice (or other measures) applicable to the local context that would improve the prevention of RTCs and related neurotrauma?	

## Methods

### Study design and setting

A descriptive qualitative approach was utilised for this study as the focus was to learn about the key informants’ perspectives rather than the generation of theories [18, 19]. As an approach that allows the researcher to remain close to the data and the surface of words and events, qualitative description allows for obtaining straight answers to issues of relevance to practitioners and policy makers where this method has been shown to be useful for developing and refining interventions [18, 20].

This study was reported in accordance with the Standards for Reporting Qualitative Research (SRQR) guidance (see Additional file 1) [21].

This qualitative study took place in Visakhapatnam, the largest city within the state of Andhra Pradesh in the south of India. This city, which is situated on the bank of the Bay of Bengal, has a population of 4.1 million and a population density of 3995 persons per square kilometre [22]. The main language spoken is Telugu, which is also the official language of the State.

This industrial city has a radial form of a road network totalling 2007 km, with 72 km of national highway that pass through the city [22, 23]. This popular tourist destination has nine arterial roads and ten junctions which are not all signalised [22]. Arterial roads are high capacity urban roads whereas junctions act as connections to the main places in and around the city [24].

As with most cities in India, the most common road users are motorised two-wheelers, followed by non-motorised two-wheelers and pedestrians [22, 25].

At present, the emergency medical services (EMS) consists of the national 108 ambulance service that provides a free emergency response and transport to nearby hospitals [26]. There are also privately-owned ambulance services which would provide transport for a fee. Currently, there are 13 trauma care centres, most of which are located in the city centre. One of the largest trauma centres is the King George Hospital, a government hospital that is a referral centre for the city, adjacent districts and neighbouring states [27]. As one of the collaborators of the National Institute for Health Research Global Health Research Group on Neurotrauma (NIHR GHRGN), King George Hospital is also one of the trauma centres with neurotrauma and rehabilitation expertise, and is the workplace of some of the participants [17, 27].

### Participants and recruitment

Participants were purposively selected to represent the following categories: commissioning stakeholders; service providers; community or local patient group/advocacy group representatives. Commissioning stakeholders would include policy makers, law enforcement officials and local government officials whereas service providers would encompass emergency service staff, rehabilitation service staff and trauma physicians or surgeons. Representatives from the community or local patient/advocacy groups are those who are involved in various groups which support patients with a history of neurotrauma or former patients themselves.

Potential participants were identified by the local research team and invited to participate via telephone. These telephone calls were made by one of the authors (KMNR), and participants were furnished with information on the overall research project, the current study and all consenting requirements.

As this study is embedded within a larger body of work (GPONT-RTC), a specific time frame was allocated for its completion. As this time frame did not allow for additional recruitment, the final sample consisted of ten participants. These were individuals who were known to be active in road safety efforts in and around the city. Some participants had been involved, in various capacities, in other research projects organised by the local research team. All participants lived and worked in Visakhapatnam.

### Data collection

Semi-structured face-to-face interviews were conducted between October 2019 and February 2020 using a standardised interview guide. The interview guide was developed in English and translated into Telegu by a member of the local research team. Back-translation was carried out by an unrelated party to ensure linguistic validity and reduce bias.

Prior to the commencement of data collection, the guide was pre-tested on two participants who were not part of the original cohort but held similar occupations to some of the key informants. Minor adjustments to wording and phrasing were made after pre-testing.

Interviews were carried out by one of the authors (KMNR). The interviewer was a male, with a background in co-ordinating neurotrauma prevention programmes for King George Hospital, trained in qualitative research, and fluent in both Telugu and English. The interviewer had previously interacted with some of the participants through these neurotrauma prevention projects.

Interviews were either in English or Telugu depending on participant preference, and were carried out at the participants’ workplaces. Participants were asked a series of open- and closed-ended questions that addressed the objective of this study. The main topic areas were: perceptions of RTCs in their city or country; perceptions on current strategies for RTCs and neurotrauma prevention, including the role of research; how prevention of RTCs and neurotrauma may be improved. Participants were also asked to share their own understanding or definitions of neurotrauma prevention.

During the interviews, participants were able to speak freely and raise additional topics relating to the issue under research at the end of their interview. All interviews were audio-recorded and lasted between 15 to 60 min.

After each interview, the interviewer would discuss the session with the lead author (SMS), and initial thoughts or impressions were noted down in the form of memos.

### Data management and analysis

Data was managed and analysed using the Framework Method. This method, which has been used since the 1980s, is an increasingly popular approach for the analysis of qualitative data, especially in medical and health research [28]. As a methodology that sits within the broad family of analysis methods termed thematic analysis, this method identifies commonalities and differences in data, and relationships between different parts of the data [28, 29]. It also emphasises how a priori issues and themes that emerge from the participants’ narratives should guide the development of the analytic framework [30]. This method is highly suited to qualitative descriptive studies as it is not aligned to any particular philosophical or theoretical approach and is useful for describing what is happening in a particular setting or context [28, 31]. In addition, the Framework method allows for the generation of straightforward, transparent results through low-inference interpretation, which is in keeping with the features of qualitative description [19, 32].

The Framework Method is divided into seven stages: 1) transcription, 2) familiarisation with the interview, 3) coding, 4) developing a working analytical framework, 5) applying the analytical framework, 6) charting data into the framework matrix, 7) interpreting the data.

In our study, analysis was carried out manually where this method was applied in the following way:

A professional translator translated and transcribed the audio-recordings into English. The same service also transcribed the other interviews that had been carried out in English. As a result of translation, the terms ‘accident’, ‘road accidents’ or ‘road traffic accidents’ are found in the quotes instead of ‘collisions’, and these are used interchangeably.

The local team then checked the transcripts for errors, and necessary edits were made. Two investigators (SMS and GDNS) then read and re-read transcripts and listened to audio-recordings of the interviews that had been carried out in English. Initial impressions were recorded at the sides of the transcripts. To increase rigour, peer-checking or investigator triangulation was carried out where the same two investigators independently performed line-by-line coding on the first three transcripts, generating inductive and deductive codes. Codes were compared and any discrepancies were resolved through discussions. Overall, there was a high level of agreement on the codes, and these were used to develop the initial analytic framework, which was used to code the remaining transcripts. Revisions were made to the original framework as new codes emerged, and finalised once the last transcript was coded.

The finalised analytical framework which consisted of categories of grouped codes, was systematically applied to each transcript where the relevant text was highlighted and assigned a category. The indexed data was summarised by category and charted onto a spreadsheet. Themes were developed by exploring patterns and relationships within the charted data, in accordance with the research objective and new concepts that emerged from the interviews.

## Results

Ten interviews were carried out with three commissioning stakeholders, four service providers and three community representatives. All participants were male except one, with age ranges between 27 and 58 years old (see Table 2).

**Table 2:** Profile of key informants

Key informant	Designation and Group	Age (in years)	No. of years involved in RTCs prevention
1	Neurosurgeon (Service provider)	44	12 years
2	Director of Non-governmental organisation (Community representative)	52	13 years
3	Nurse (Service provider)	30	4 years
4	Local government official (Commissioning stakeholder)	43	13 years
5	Ambulance service staff (Service provider)	27	2.5 years
6	Law enforcement official (Commissioning stakeholder)	55	25 years
7	Rehabilitation service staff (Service provider)	34	8 years
8	Law enforcement official (Commissioning stakeholder)	58	10 years
9	Former patient and patient advocate (Community representative	44	2 years
10	Former patient (Community representative)	40	2 years

Five themes emerged from the analysis of the interview findings: participants’ knowledge of RTCs in their city, state and/or country; impact of RTCs; current preventative strategies; barriers to prevention; perceived recommendations for improving prevention. The summary of the themes with categories and descriptors can be found in Additional file 2.

### Participants’ knowledge of RTCs in their city, state and/or country

#### The nature and extent of the problem

Participants recognised that RTCs are a major problem in their city, state and country where most collisions occur among adolescents and young adults, and involve pedestrians and two-wheeler users:

*“India has the highest road traffic victims, and moreover this year Andhra Pradesh has a 10% increase in road accidents compared with last year, and 70 to 80 percent are pedestrians and two-wheelers.. At present there are 97 accident prone areas in Visakhapatnam. The collisions are in those between 18 and 40, youth people [sic].”.(*Key Informant 2)

Most participants were also very clear about the role of RTCs in neurotrauma causation and felt that RTCs are the leading cause of neurotrauma and that most casualties will sustain injuries to the head injuries. However, one participant presented a different view:

*“In Road traffic Accidents head injuries are less than other injuries like leg and hand fractures which are more and head injuries are very less [sic].”* (Key Informant 10)

#### Factors and risk factors leading to RTCs and RTC-related neurotrauma

Participants identified various human factors that led to RTCs and RTC-related neurotrauma, the most common being drink driving and speeding. Many also discussed the lack of or improper use of personal safety equipment, namely helmets:

*‘Most of the traffic accidents and brain injuries are caused in two wheeler bike riding [sic].. Most of the riders do not wear helmet or they do not wear it properly [sic].”* (Key Informant 8)

*“ … people carry the helmet on the bike without wearing it; it is safe if they wear it.* “(Key Informant 3)

Other frequently observed traffic rules violations included wrong-way driving and using mobile phones when driving. Another issue that was raised was underage driving.

*“Nowadays more accidents are happening because of minors riding vehicles. People should not give their vehicle to others who do not have license [sic]”* (Key Informant 5)

Some participants felt that poor road infrastructure and design was a common environmental factor that contributed to this problem.

*“Roads everywhere have pot holes, unfinished works of sanitary, water and other departments after they dig for various repairs and renovations, making it risky for the bike riders.. and increasing the chances of skidding and falls. When riders fall the first impact would be on head leading to head injury [sic].”* (Key Informant 9)

*“..This (highway) stretch it is totally in the center of the city where all types of people are moving -pedestrians, old, children, this, that [sic]. If you have lorries going, say, 70 kilometres per hour, we have to use stoppers (to prevent collisions) and all this will cause more damage than the open highway where there is no village or no public there, and the lorry driver can go 70 to 80 kilometres per hour- nothing will happen.”* (Key Informant 6)

Bad weather conditions were reported to also contribute to poor road infrastructure, in addition to causing poor visibility, especially for those on two-wheelers.

Other environmental factors that resulted in RTCs included being hit by the opposite driver or by moving obstacles that suddenly emerge onto the road. One participant narrated:

*“Obstacles like dogs, pigs and children on the road are also causing accidents. I am a bus mechanic. That day one bus is in repair, so I had gone to the workshop on that morning 5 am [sic]. From the side of the depo, one forest pig came in at high speed and hit my two- wheeler, then I fell down unconscious [sic]. After that I was admitted in hospital.”* (Key Informant 10)

Vehicle and equipment factors were brought up by some participants when discussing RTC and neurotrauma causation. Overloaded vehicles were sometimes seen on roads and highways, as reported by the following participant:

*“Auto, lorry and even buses when they were over loaded above the capacity of the vehicle and it will automatically give more pressure to the driver [sic]. The vehicles will be uncontrolled and the vehicle will fall down from bridges or fall down into rivers, hit the road dividers and so on [sic].”* (Key Informant 1)

Another participant talked about the widespread use of substandard safety equipment that did nothing to protect from RTC-related neurotrauma, but was simply to appease law enforcement officials:

*“..even if they (two-wheeler riders) use helmet, they use a sub-standard helmet as it is cheap and can escape the fines imposed for not using helmet by police [sic]. These cheap and low quality helmets break easily causing severe damage to their heads leading to skull fractures, brain injury and internal bleed [sic].”* (Key Informant 9)

### Impact of RTCs

Participants agreed that RTCs can lead to death and result in injuries. Of all the injuries sustained in RTCs, neurotrauma was the most common and devastating, where this was illustrated in its health impact on casualties.

*“Road accidents majorly lead to head injury [sic]. (Other injuries) all are generally curable, treatable and small. The head injury cases are mostly unable to cure completely [sic]*.” (Key Informant 3)

One participant explained how the increase in RTCs meant an increase in neurotrauma cases, which in turn was overwhelming the current healthcare system, to the detriment of the casualties.

*“Mostly these major injuries (from collisions) are head and spine [sic]. So now we can see the figure is so huge so the need of centres, the need of hospitals, trauma care, emergency care are most important. We are having only (few) beds that are available so most (neurotrauma cases) are without any sort of rehabilitation [sic]*.” (Key Informant 2)

RTCs also had an economic or financial impact particularly on families as most of the casualties would be the only earning member of the family. Participants described not only the loss of income, but the fact there was no system in place to provide support or assistance for households who have lost their sole breadwinner.

*“The Impact is more on their families because most of the times victims of RTA (road traffic accidents) are the bread winner of the family and leading to financial crisis [sic]. There is a Telugu saying that goes ‘if an accident happens it is not the individual that is affected, it is the family on the road (hopeless) [sic]’ “(*Key Informant 9)

Some participants mentioned the economic impact RTCs would have on the country*:*

*“A lot of good professionals we are losing in the city [sic]. They are very good in work and different professionals. We are losing such a great people in the city due to these accidents [sic].*” (Key Informant 6)

*“As per the statistics of government of India these accidents are affecting around 3 % of our GDP* (Gross Domestic Product)” (Key Informant 4)

One participant discussed the social impact of RTCs, bringing up an interesting point:

*“(These accidents) bring a bad name to the city, that this is very accident- prone city in this area. So for that lot of work has to be done to control such type of accidents.”* (Key Informant 6)

### Current preventative strategies

#### The role of the government and related organisations

Participants identified the various rules and laws that were in place to address the issues of drink driving, speeding and unauthorised use of motor vehicles. Many mentioned regulations relating to helmet use and how it was also compulsory to purchase a helmet when purchasing a new motorcycle.

Participants felt that enforcement of this legislation was in place where police would carry out checks for drink driving using breath analysers, and spot checks for valid licenses. The police were also carrying out speed enforcement, not only through road-side patrolling but also using technology:

*“We are taking lot of steps like regularly we are keeping our (police) to keep watch and check the over-speeding [sic]. We have laser guns in the city, 10 more we are going to procure, so everywhere we are keeping these laser guns and we are monitoring the movements of those people [sic].”* (Key Informant 6)

This enforcement also allowed for offenders to be booked and issued with penalties and high fines or imprisoned, if their offense was serious enough. An example of this was seen in relation to those who violated the helmet laws:

*“We have many helmet cases, those persons who are not wearing, we are taking photographs and sending chalans (citations), and all then now these people will start wearing helmets and start following the traffic rules [sic].”* (Key Informant 6)

Many participants brought up how the police were involved in prevention not only by taking action against rule breakers but also through educational and awareness efforts:

*“Definitely to prevent road traffic accidents traffic police are organizing so many awareness campaigns to prevent all these things.[sic].”* (Key Informant 7)

*“(The police) have started awareness programmes in the colleges and educational institutions.. (They) are going and creating awareness among the students, that you should follow the traffic rules, and that time (they) are showing some incidents where people died.*” (Key Informant 6)

Another law that was brought up by many participants was the Good Samaritan Law which ensured that members of the public who assisted RTC casualties were given the necessary legal protection from harassment by the police, judiciary system or the healthcare system.

In the light of this, participants discussed programmes where volunteers were trained to respond to RTCs:

*“(The police) are launching tomorrow one programme called “road safety friendly”. We are starting where we have trained almost 50 volunteers especially shop keepers and the workers all over the high way. Every one kilometer we have trained them and 150 of our traffic constables and local police who are working on the highway if any accidents happens they work like first responders immediately. They will provide first aid for them (victims). We are providing them (volunteers) first aid kit and immediately they will inform to 108 (ambulance)[sic]. They will take local assistance [sic] and shift the fellow to the nearest hospital and we will make them aware which are nearby hospitals, whom to contact, how to contact, how to shift the fellow because in accident multiple fractures are there, spine fracture are there. How to tackle such situation, we have trained them [sic].”* (Key Informant 6)

Further on the Good Samaritan Law, the government and healthcare organisations also have taken it upon themselves to educate and create awareness among the public about this law:

*“The DMHO (District Medical and Health Officer), District collector, KGH (King George Hospital), have taken it into consideration and we have put the posters (about the Good Samaritan guidelines)- to help the crash victims without any fear [sic].”* (Key Informant 2)

Strategies relating to vehicle engineering were mentioned by a few participants, where these were the compulsory installation of in-vehicle speed management devices in new vehicles and commercial vehicles including school buses. The road engineering strategy identified by participants was the presence of speed bumps, or speed breakers, on roads.

Some participants mentioned road safety committees and felt that these are an instrumental component of prevention:

*“Government is constituting ‘district road safety committee’ in every district. District collector is the chairman of this committee and deputy transport commissioner is the convener, along with them DMHO/DCHS (District Medical and Health Officer/District Child Health Services), superintendent of district hospital, officers from R and D (Research and Development unit), Panchayati Raj (Rural development) department are coordinating with each other and identifying the causes of road traffic accidents and remedies [sic]. The committee meetings happen periodically and discuss the strategies to reduce the road traffic accidents, to prevent neurotrauma injuries*.” (Key Informant 4)

*“We have gone to attend the meeting of state road safety committee and we have raised so many issues so now they are empowering the enforcing the law [sic]..*” (Key Informant 2)

#### The role of individuals and communities

One issue that emerged was how preventative strategies were developed and implemented not only by the government, but also by individuals and communities. An area where this was identified was through education and awareness efforts:

*“I personally go weekly thrice [sic] for counselling the drunken drivers and for my way we also counsel the employees of IT (Information Technology) and ITES ( Information Technology Enabled Services) companies. So try to educate them.. I have started another banner called project ‘Bhavishya’. This Project ‘ Bhavishya’ is three ‘R’s. First ‘R’ is road safety awareness. Second ‘R’ is right to emergency care of the crash victim. Third R is for rehabilitation of the crash victim.”* (Key informant 2)

Some of these measures were carried out in collaboration with government entities:

*“I have conducted several awareness programs in my college and near- by schools and colleges..with collaboration from [..] police, I conducted several campaigns about prevention on [sic] road traffic accidents*. “(Key Informant 9)

Aside from these formal programmes, many participants brought up how education and awareness could be delivered informally through communicating with the people they crossed paths with in their jobs and daily lives:

*“As a surgeon when a patient arrives with traumatic injury I try to understand, first of all what is the cause of the accident. After knowing that what is the reason, like is it due to drinking of alcohol or over speed driving or due to the fault of other driver, I try to motivate the patient to avoid same mistake again [sic]*. “(Key Informant 1)

*“My role is to make my friends, family members and all known people aware of these head injuries, and the possibilities of head injuries if they go over bike without helmet the chances of head injuries are more. And I always inform to people to make journey safer with enough speed [sic].”* (Key Informant 5)

Another area where there appeared to be community involvement was in the area of tertiary prevention of neurotrauma where one participant mentioned a rehabilitation centre that was built by a community-based organisation which offers free services for RTC casualties with neurotrauma.

#### The role of research

Many participants described the different types of ongoing research that was being carried out by different entities and how these had a role in prevention of RTCs and neurotrauma:

*“Some research is going on at New Delhi. Because we will come to know whether it is severe road accident or moderate or simple injuries and how many people died, how many people have memory loss, how many people suffering with disabilities, what is the effect on the family etc.[sic] Because of this research we are getting the information about how many people dying in the road accidents, how many people and families are suffering due to road accidents and what steps we should be taking in order to prevent this [sic].”* (Key Informant 1)

Some discussed the research that they are involved in:

*“I am involved in few researches [sic] in Traumatic Brain Injury. We work on psychosomatic problems of the patient after Traumatic Brain Injury and we work on involvement of transcranial magnetic stimulation, transcranial direct current stimulation which help in outcome of the motor control issues and use of the role of neuro plasticity in traumatic brain injury recovery, we work on few researches [sic]. Whatever research is going behind the system [sic], (we) will always give the information in accurate way so that we can assess the severity of the problem, so according to that we can take the preventive measures.”* (Key Informant 7)

#### Effectiveness of current strategies

A few participants brought up how they felt that the current strategies were effective in reducing RTCs and related neurotrauma as the government appeared to consider this an important issue:

*“Government of India is also doing a lot of work and spending money on road safety by involving all the state governments. Even the Supreme Court committee in road safety is also reviewing the situation with state governments to reduce RTA’ s (road traffic accidents). These steps are helping to reduce accidents*.” (Key Informant 4)

However, one participant felt that the strategies were useful to some extent but there were still areas that were lacking.

### Challenges to prevention

Participants raised many factors that appeared to affect the prevention of RTCs and neurotrauma. Some brought up physical factors such as overpopulation and overcrowding and its impact on road use.

*“In India 80% of the roads are with heavy traffic and to control this rush of vehicles it is very difficult for police authorities [sic].”* (Key Informant 9)

Others discussed the lack of funds and manpower for carrying out preventative strategies. This was particularly relevant in the area of research and the trauma system, and especially in the area of law enforcement:

*“You can see this city has more than 30 lakhs (more than 3 million) population where we have only 3400 police man, in that only 500-600 working in the traffic (division). Remaining are busy in some other duties [sic]. We can say it is totally disproportionate..hardly 600 people can’t control the 30 lakhs and more than 8 lakhs vehicles are in the city [sic].”* (Key Informant 6)

One participant also felt that the police had a lot of other responsibilities, especially during certain times of the year, and that there was not enough time for traffic law enforcement.

This lack of resources was felt to be one of the contributing factors which led to gaps in the current preventative strategies. However, participants also felt that there were other flaws, particularly inconsistent implementation:

*“Generally what happened we are creating awareness only in the educational institutions, limited areas where public is less, but large numbers of people are dying on the rural areas and illiterate people, so they are not getting this type of counseling or training..[sic] Conducting one day, two days rally will not make much difference [sic]. Of course people will come to know that these are the rules. It should be like a part of a habit [sic].”* (Key Informant 6)

*“Trauma care facilities are available at very few locations now. Our facilities are concentrated in certain centralised locations only. Due to present centralised systems, it's difficult for reaching the centre from out skirts in Golden hour [sic].”* (Key Informant 4)

Building on the issue of inconsistent implementation, one participant explained how laws were not being enforced consistently or equally:

*“Police people also not enforcing the law at all the places in same way [sic]. In some places they are using strict rules and where in some places they are not serious about the issues, then people are not taking these things seriously [sic]. They are taking very easily about rules and regulations of the traffic [sic].”* (Key Informant 9)

*“Nowadays the fines are hiked [sic], but the people who are having political background and not following rules are not being fined.”* (Key Informant 3)

A lack of appropriate action was cited as another factor that was a barrier to the prevention of RTCs and neurotrauma despite there being strategies in place. Participants felt there were insufficient awareness programmes and lack of enforcement or implementation of laws. The lack of root cause analysis was an example mentioned by some participants:

*“If accident happens between car and bike, immediately people blame the car vehicle people only [sic]. Even sometimes if two-wheeler hits the stopped car also people blame on car vehicle people only [sic]. Police people do this wrong [sic]. Generally, whenever accidents occur root cause for the accident is not sought, rather they blame the big vehicle and find fault always without properly investigating the case to find the proper cause [sic].”* (Key informant 9)

One participant narrated how this lack of appropriate action was also seen in the current trauma system:

*“Here there is another issue happened after meeting accident [sic]. Immediately people shift (the victim) to PHC (Primary Health Care facility) or some small hospital. There they take a lot of time. They (the hospital) don't have scans and all these things … I will give example. Two to three days back there was an accident. Simple external injuries were not there, but he got brain haemorrhage and he was simply sent to some hospital [sic]. They gave some first aid, put some ointment and all [sic]. They have not done scan and all these things. After 2 days he developed complications and died.”* (Key Informant 6)

Other participants found this was also true in relation to the implementation of road engineering interventions:

*“Here at my place 6 lanes widening of highway road construction work is going on [sic], and the construction material is unloaded in an inappropriate manner and safety precautions are not taken, which is increasing the chances of road accidents.”* (Key Informant 10)

*“Due to rains [sic] the holes are coming on the road, and these holes causes the accidents but they (the government) are not attempting the repair works on time [sic].”* (Key Informant 5)

In the area of research, the participants identified a lack of timely data collection, data sharing and overall scientific research. In addition, they felt that research participants tended to provide incorrect data, as explained by one participant:

*“The biggest barrier to this type of research is that people will not give proper information. For example, I had an accident and supposedly I was amputated of hand and leg, and when people enquire about the accident and the reason how it happened, general tendency is to avoid giving appropriate answers to these questions [sic]. If we are unable to get correct data we cannot come to a proper conclusion about any research [sic].”* (Key Informant 9)

Poor governance was also cited as a factor that had a negative impact on the success of current preventative strategies where many participants reported the issue of corruption:

*“The corruption by police is another barrier and they collect fine charges from the areas which are crowded … Corruption is the one in the present law system [sic].”* (Key Informant 3)

*“(Police) are threatening bikers and taking bribes, and leaving them (offenders) unpunished.”* (Key Informant 10)

Aside from this, participants felt that there was a lack of political will which resulted in poor procedures and infrastructure, and the lack of coordinated efforts between government agencies. There was also the problem of obstacles from opposing political parties.

Another area which participants felt challenged successful prevention of RTCs and neurotrauma was the mindset and behaviour of the people. Road users tended to disregard traffic rules due to a general lack of concern for road safety or thinking that nothing will happen to them. Even if they did follow the rules, it was done simply to avoid fines and not for safety and avoidance of injury. This indifference was also seen when people would forget to use personal protective equipment like seat belts or helmets, or consider it unnecessary when travelling short distances.

Some participants brought up how the mindset and behaviour of parents affected the safety of their children:

*“Another important thing is parents, they are purchasing new vehicles and giving the vehicles to minors, is also big problem [sic].”* (Key Informant 9)

*“Children are playing on the roads and their parents are not caring about them is also causing more accidents [sic].***”** (Key Informant 10)

The participants felt that complacency was also evident in the way vehicles were portrayed in the media:

*“The motor vehicle manufactures in their advertisements and in the movies and TV channels promote their vehicle’s high speeding capacity and entice with thrilling and action promos, causing a huge impact on youth [sic].”* (Key Informant 9)

Participants felt that even the entities involved in prevention did not have the right mindset and behaviour and that this would have an influence on the general public, as narrated by the following participant:

*“These police people [sic] should be like a role models to the society. Police people [sic] are not using helmets. They are using cell phones while driving. With these all [sic], people are not following rules. First, police people [sic] should follow these rules and regulations properly then they can ask others to follow.”* (Key Informant 10)

Lack of education and awareness among the public were identified as factors that not only influenced mindset and behaviour but also affected the success of preventative strategies, especially newly implemented ones:

*“Education level is very low. People are not aware regarding the traffic rules.. they are not aware what are the changed rules [sic].”* (Key Informant 6)

### Perceived recommendations for improving prevention

Participants provided suggestions on how to improve the prevention of RTCs and neurotrauma. These appeared to be linked to changes in policy and behaviour.

#### Enhancing current strategies

i. Education and awareness

An important area that was felt needed improvement was in educating the public in RTC and neurotrauma awareness, identifying a need to target RTC-prone groups or areas where RTCs were high, and carried out more consistently:

*“Most important thing is that to create an awareness we should select the targeted persons who are more probable to get such type of accidents, like students because they are young [sic]. They don’t know traffic rules and regulations. We should create awareness more on the targeted groups like I found here in that most of the deaths are on two wheelers [sic]. We should target on these two -wheeler drivers, whether they are students or some of the employees, so we should target them [sic].”* (Key Informant 6)

Many felt that education should be started from an early age where road safety should be introduced as part of the school syllabus for primary schools, and continued in secondary schools and colleges. In addition to creating awareness amongst the children and adolescents, this would also create opportunities for education and awareness of households in general:

*“There must be road safety syllabus for school and college students. It should be implemented in schools and colleges. If school and college children are properly educated in this awareness programme, they will be the primary step in their family to avoid accidents [sic].*” (Key Informant 2)

Participants also discussed different ways these efforts could be made more effective and far-reaching, for example through road-side events, getting former casualties or their families to share experiences, and through the media. One participant also highlighted how the education or awareness sessions should be carried out in a way that is understood by the people, especially in villages amongst those with low educational attainment.

Aside from the public, many participants also felt that education was also a necessity among government bodies and other entities involved in prevention through regular and relevant training programmes.

ii. Research

Besides being educated about road safety and neurotrauma, participants felt there needed to be more awareness about previous and ongoing research amongst the government and any agencies involved in prevention as well as the public. Research findings should be disseminated to all these groups either formally or through social media, depending on the target audience. One participant talked about the role of celebrities in the dissemination of research findings.

Participants felt that increasing awareness on research could attract other researchers to work on this issue and enable innovative solutions to be developed with policy-makers.

When discussing the practical aspects of how current research could be improved, participants felt that research into the causes of RTCs and the number of casualties, as well as on pre-hospital care should be prioritised. Research should also be conducted in more centres in both cities and peripheral areas, and should involve former casualties. It should also be carried out consistently by a dedicated group of qualified people and should be regularly reviewed or audited to ensure accuracy of procedures and findings.

iii. Pre-hospital and trauma system

Many participants felt there was a need to improve the current first responder system where there was a need for more layperson involvement:

*“In any society the first responder is the public. They can’t depend on the police. If something happens also people should respond immediately [sic]. They should behave like a first responder, providing first aid, give the injured person comfortable position and call the nearest police station, ambulance [sic].”* (Key Informant 6)

“If we train the RTC (Real Time Clock)* drivers and the auto drivers how to help

*crash victims, it may be CPR (Cardio-Pulmonary Resuscitation), it may be BLS (Basic Life Support), I believe that will be our strategy because auto rickshawwalas (auto drivers) and RTC drivers, lorry drivers, if given proper education in this, they can easily handle the patient [sic].”* (Key Informant 2)

#### *A real time clock or RTC driver is a bus driver

One participant mentioned the possibility of building small first aid centres in villages so casualties in rural areas would have an immediate and dedicated space where they can receive care while waiting for transport to hospital.

There was also a need to improve ambulance services through proper training of staff and ensuring availability of appropriate medical equipment within the ambulance. The response time should also be reduced to under 10 min, where this could be achieved if all the ambulances were part of a network.

Some participants discussed the need for more trauma centres particularly with expertise in neurotrauma. This not only improved access to care but also mitigated the issue of casualties being transported to the nearest healthcare facility which may not have the necessary expertise to deal with their injuries. There should be coordination between the hospitals and ambulance services so that the ambulance staff would be aware of centres which provide different facilities, such as neurotrauma care, so that casualties could be transported to a healthcare centre where they could receive appropriate management regardless of whether it is private or government institution.

Decentralisation of trauma facilities was brought up as another way to improve access to care:

*“Decentralisation is important. If we decentralised them we can also save many lives. Providing trauma care facilities in the outskirts of the city. Trauma centres should be established in industrial corridors, and upcoming urban centres, highways and peripherals of the city [sic] which will help treat neurotrauma immediately and can save lives.”* (Key Informant 4)

iv. Enforcement and legislation

Participants felt that there was a need for stricter enforcement of laws and that penalties should be implemented fairly and uniformly. An audit system was suggested as a way to ensure enforcement was carried out properly and consistently as well as a programme to identify accident-prone areas which would allow for a focus of enforcement in those areas.

A few participants felt that the penalties should be harsher and that even small faults should be punished with high fines or imprisonment to ensure people would not want to violate the traffic rules. Some suggested that traffic sign posts should also be increased at every junction to reduce RTCs.

Some participants discussed how legislation changes could influence the area of drink driving:

*“Additional efforts will be like taking strict action against drunken driving [sic] by eliminating liquor shops near the highways.*” (Key Informant 4)

Several participants also felt there should be proper implementation of the Good Samaritan Law:

*“I believe that (accidents) can be prevented if .. the good Samaritan guidelines which are given by the honourable Supreme Court can be implemented in the grass root level [sic].”* (Key Informant 2)

One participant talked about changing the police system without changing the rules. He explained:

*“Change of police system and genuine implementation of the existing rules and regulations should be done, everything will fall in place..there is no need of new rules and regulations in this manner [sic]. New regulations will give some more headaches and problems to the society.*” (Key Informant 10)

v. Road engineering

The participants felt that the current status of the roads needed to be improved not only by improving the quality and width of roads but by ensuring roads were constructed safely and with care. They also explained how roads should have more than one lane to cater for vehicles with different speed capacities.

There should also be a system in place for the reporting and immediate rectification of any road defects.

Road engineering efforts should also focus on speed reduction, for example having more speed breakers at junctions between small roads and main roads.

#### Establishing collaborations and partnerships

Some participants mentioned the need for inter-and intra-agency collaborations and partnerships, as well as partnerships between the public and private sectors.

*“I feel strongly the main stakeholders are four: one is police department i.e traffic, second is health department i.e. DMHO, third which is very important aspect is the transport department where they have logistics how to implement strategies and fourth one is volunteers who are NGOs (non-governmental organisations)[sic]. They should come forward to coordinate with all these three departments then only (preventative strategies) will be successful.”* (Key Informant 2)

Another important and necessary partnership was between the government and members of the public:

*“First thing that we should take the help of the public [sic]...they should also keep watch, they should also come forward and assist the police. Whenever such things are happening, they should bring to our notice. If they found some other people, if they notice that people are violating the traffic rules, immediately they should call we have 100 number and 109 like that [sic]. If they take assistance [sic] definitely it will be reduced.”* (Key Informant 6)

#### Changing mindset and behaviour

Participants believed that the prevention of RTCs and neurotrauma could be improved if the mindset and behaviour of the citizens were changed. All traffic rules needed to be followed with the understanding that it is for their own safety and not just avoidance of punishment. Abiding by the rules would also lead others around them to do the same. The public should also realise that they have a bigger role to play in the prevention of neurotrauma and RTCs, for example by organising awareness campaigns for their community and providing first aid to unknown RTC casualties, as narrated by one participant:

*“Rather than depending upon the Government, we ourselves should do something from our side, then only (prevention) can be improved a lot.”* (Key Informant 2)

It was felt that there needs to be a strong political will with governmental support for all preventative efforts. Government officials and all those involved in prevention should also set good examples to be emulated by the public in order to have a bigger impact:

*“These police people [sic] should be like role models to the society.. first police people [sic] should follow these rules and regulations properly, then they can ask others to follow.”* (Key Informant 10)

*“If all the government institutions whether semi government, Central government or State government, if they all follow good Samaritan guidelines there will be huge impact]sic]***.”** (Key Informant 2)

## Discussion

This study sought perspectives of key informants from Visakhapatnam on the local context surrounding RTCs and related neurotrauma. To our knowledge, this is the first qualitative study that also explores the issue of neurotrauma prevention in an LMIC setting, using the key informant technique.

Participants felt that RTCs are an important problem in their city, state and country, and a major cause of neurotrauma. This is in keeping with recent literature which indicate that India is a country with very high rates of RTCs, contributing to more than 60% of neurotrauma admissions [6, 33–35].

Identified human factors that resulted in RTCs and neurotrauma were those commonly seen globally, namely speeding, drink driving and not using personal safety equipment [33].

Given the fact the main vehicle type in India is the two-wheeler, there was a lot of discussion surrounding helmet use [6, 25]. Participants noted that many riders and passengers would not use helmets, and even if they did, it was not done properly. Although India’s current helmet law meets best practice, several local studies have shown that helmet use in the country is generally low and varies from state to state [36–39]. Although there have not been any qualitative studies to specifically explore the reasons behind this, some local publications have indicated that the lack of adequate law enforcement and ignorance about the need for a safety helmet tend to result in the failure to use helmets, or use them in the correct manner [38, 40]. These factors were also identified by participants as barriers to the successful prevention of RTCs and neurotrauma.

The purchase and use of helmets which were of poor quality was another problem, where this was often done just to appease law enforcement officials. In India, helmet standards are regulated by the Bureau of Indian Standards (BIS) where approved helmets would have the Indian Standards Institute (ISI) mark [40]. Nevertheless, the sale of non-standard helmets is prevalent, and many two-wheeler riders who tend to be from the lower socioeconomic class would opt for this poor- quality equipment due the lower price and wider availability [40, 41]. As a result, this group becomes more vulnerable to neurotrauma [41].

Poor road engineering, in particular poor road quality was cited as another factor contributing to the rise of RTCs and related neurotrauma. This is not unusual in LMICs where several studies, especially from the African region have identified dismal road conditions as a cause of RTCs [42–46]). In the Indian context, a qualitative study done in Hyderabad also reported similar findings when participants identified poorly constructed, damaged and poorly managed roads as a reason for poor road safety [6]. Again, this appears to be a common problem in India which arises due to outsourcing of road construction to contractors who tend to construct roads poorly to facilitate new contracts to reconstruct or repair the road [6, 47]. In addition, maintenance practices are not carried out in a uniform manner [48]. This is consistent with our findings where participants reported that roads were not being repaired on time, with no system in place for reporting road defects.

Another area of concern was the poor design of roads, particularly highways. Reports have shown that the planning and construction of these highways are often haphazard where there are not always clearly defined lanes that segregate slow moving traffic from fast moving traffic, and often merge with urban roads [49, 50]. As a result, highways end up carrying about 40% of road traffic at a time although these only constitute 2% of the total national road network [51]. In addition, LMICs such as India face issues such as population density and different levels of motorisation which make it more challenging to utilise global road engineering solutions [52, 53].

Although there is an overall lack of road engineering interventions as well as studies assessing the impact of such interventions on RTCs in LMICs, participants were well aware of its importance in preventing RTCs [52, 54]. Most of the suggestions for improvement provided by the participants have been recommended by experts in road safety and engineering, namely increasing the width of roads, having different lanes separating different vehicles with different speeds, and speed control through speed bumps or speed breakers [54–56]. Another suggestion from the participants was to implement a system for reporting and immediate rectification of road defects. This is also something that has been recently raised as a recommendation by a Joint Working Group for Road Safety,and would be instrumental in strengthening and improving the pre-existing Road Safety Audit which was put in place by India’s Ministry of Transport and Highways to evaluate the safety of an existing road or infrastructure, and to identify safety concerns of a proposed infrastructure [7, 57, 58].

Moving obstacles on the roads also emerged as another cause of RTCs. Some LMIC studies, including from India, have identified animals on the road as a contributor to the problem [59–61]. This is especially dangerous for two-wheeler riders and passengers who tend to be most vulnerable, where sudden encounters with these obstacles, usually in rural areas, often lead to injury, as experienced by one of our study participants [62, 63]. Nevertheless, the lack of precise data on the how much moving obstacles lead to RTCs warrants further research [59, 60].

Given the close relationship between RTCs and neurotrauma, participants illustrated how there were long term implications in terms of recovery from injury and the capability of the current healthcare system to manage these patients. Recent publications from India have indicated that there is a shortage of neurosurgical expertise and facilities in India as a whole [64–66]. In addition, most of the centres that offer neurosurgical care tend to be concentrated in metropolitan areas and larger cities [66, 67]. As RTCs in India tend to occur mostly in rural or peri-urban areas and along highways, the centralisation of services poses a problem with access, where many casualties do not reach the appropriate healthcare facility within the Golden hour [6, 39, 68]. This was highlighted by participants where decentralising trauma services and establishing facilities in the outskirts and along highways was suggested as a way of addressing this issue.

Participants also identified RTCs as being more common among adolescents and young adults. Studies have shown that up to two-thirds of injuries and deaths from RTCs in India are reported in those between the ages of 15 and 40 years [10, 34, 68, 69]. This group includes the most economically active population, most of whom are males [39, 69, 70]. These findings are in keeping with the results from our study where participants described the economic impact of RTCs on families and the country as a whole through the loss of this economically productive group.

Another issue raised was the lack of support for families who had lost their sole breadwinner to injuries or death from an RTC. A case study carried out in Bangalore, another city in India, showed that affected households tended to resort to borrowing money or selling assets to cope not only with the loss of income but with the cost of caring for the injured family member [71]. Less than 20% received any compensation from the government, employees or insurance companies [70]. This is a common scenario in India, where many families end up spending more than 10 times their monthly income for the immediate and long-term management of the RTC casualty [72].

Recognising the seriousness and complexity of this problem, the government has been attempting to make progress towards reducing RTCs and RTC-related injuries through various initiatives, the most recent being the enactment of the Motor Vehicle Amendment Act [7]. The 2019 amendment to the Motor Vehicle Bill has included several new policies and regulations addressing issues of sub-standard helmets and vehicles, and increasing penalties for violation of traffic rules and even failure to comply to road design standards.

Another area that is addressed in the new amendment is underage driving. A recent study showed that in 2015, 45,000 RTCs occurred among minors without driving licences [39]. From our study, this was highlighted as a problem that seemed to arise when adults, especially parents allowed their underage children to operate motor vehicles. In the amendment, a new regulation was instituted in that charges and punishes the parent guardian or owner of the motor vehicle in the case of a juvenile who does not possess a valid licence who commits any traffic offense [10].

The amendment also discussed the Good Samaritan Law, that was introduced in 2015 as guidelines to safeguard bystanders who help trauma casualties [39]. According to this law, the Good Samaritan is not obliged to provide personal or incident details, and is protected from coercion or harassment by the police or hospital staff or from bearing the cost of treatment of the casualty they assisted [39, 73]. However, local surveys show that nearly 60% of Good Samaritans are still being harassed, and that action is not being taken against those agencies that did not comply with this law [73]. Thus, the Good Samaritan Law is now included in the Motor Vehicles Act where it has been made explicitly clear that Good Samaritans are not liable for any civil or criminal action relating to the injury or death of an RTC casualty, and should they be intimidated by any parties, strict disciplinary action will be initiated by the government [7, 74].

Interestingly, these same surveys also showed that nearly 85% of the public are unaware of the Good Samaritan Law [73]. This was somewhat reflected in our findings where there were ongoing efforts by the government to increase public awareness concerning this law. However, one concern that emerged from this study was that the public were not only ignorant about this law, but also road safety laws in general. This situation is not uncommon in India where several studies have shown that many road users were unaware of traffic rules, laws and symbols resulting in unsafe behaviour on the road [38, 40, 75]. Although road safety awareness and education programmes are being carried out by the government and other organisations as a way of rectifying this situation, participants pointed out that efforts are often inconsistent and not targeted. They felt that these strategies could be improved by creating awareness amongst those who were more likely to be involved in RTCs and carried out on a regular basis, with constant reminders through roadside events or advertising, or whenever a vehicle was purchased.

Participants also discussed the role of the media in prevention, particularly how social and support media could be used as a means of educating the public and creating awareness about road safety and traffic rules. Some participants also brought up the negative impact the media seemed to have, especially on young people through advertisements or movies that promoted speeding. This was also a similar finding in another qualitative study from India where the movies and media were blamed for their influence on driving behaviours of young people who tried to emulate high-risk behaviours seen in advertisements and movies [6].

It was therefore not a surprise that participants also felt that education and awareness should start from a young age and suggested that road safety should be part of the school syllabus for both primary and secondary schools, and how children would be able to bring awareness and education to their families. This concept of children as agents of change for family health has been seen and applied in a broad range of health-related community interventions [76]. Although studies relating have been limited with mixed results, this concept has been shown to be positive in educating and influencing behaviour change in parents [76–78].

Education and awareness efforts also appeared to be carried out by many participants in our study either as formal community organised programmes or informally. The other area where there seemed to be community involvement was in the tertiary prevention of neurotrauma with the establishment of a community-run rehabilitation centre. This is an important effort as rehabilitation services in India are limited and often not accessible [39, 79]. Community-based services provided without charge would be of great benefit to the majority of RTC casualties who reside in rural areas [39].

These findings suggest that participants believe that prevention of RTCs and neurotrauma should be the shared responsibility of both the government and the community. It was clear that prevention could be improved if the public worked together with the government. Similarly, collaborations between health, transport, law and even education sectors are thought to be important for improving prevention [6, 72]. Our results show that one of the ways this was being carried out was the establishment of district road safety committees with representation from multiple stakeholders.

Research was another area where there appeared to be ongoing efforts ranging from epidemiological-type investigations to projects involving technology. Nevertheless, participants still felt there was a lack of scientific research and issues with the conduct of this research, including imprecise data. As a result, suggestions were made for regular audits or reviews to improve research standards and to advertise research efforts to attract more research expertise. Priority areas for research identified included investigating the determinants and geographical distribution of RTCs.

Prehospital care was also another area of research that participants felt was lacking. This is interesting in the light of the fact that India lacks a systematic pre-hospital or a formalised trauma system [10, 39, 67]. Our findings illustrated the common scenario in India where RTC casualties would be transported by bystanders in private vehicles, without waiting for the police or government ambulances, to one of the nearby primary health care centres. These centres, though many and usually found in rural areas, are incapable of handling trauma as these are very basic facilities which are usually manned by a medical officer and paramedical staff [60]. Casualties would also have not received any first aid at the site of injury, even if EMS were called as ambulances are only used to transport the casualty and ambulance staff are inadequately trained to provide even basic pre-hospital care [39, 80]. In addition, the EMS in India are fragmented with private and public companies offering varying services that not always accessible throughout the country [80, 81]. Many people do not even know the numbers to call in case of an emergency, even the nationwide free telephone number for emergency services, 108 [81].

Realising this, participants suggested solutions such as integrating the EMS and ensuring ambulances are equipped with at least first aid capabilities and trained paramedics. Involvement of lay first responders or bystanders appeared to be another area of interest for participants where such a programme had recently been introduced in Visakhapatnam. Research has shown that trained layperson first responders can improve trauma outcomes particularly where prehospital evacuation times are long, which is often the case in India [23, 39, 82]. In addition, participants also felt that this would demonstrate community participation, part of the joint responsibility towards prevention.

Although participants appeared to be in support of the current governmental efforts in preventing neurotrauma and RTCs, they still raised some issues which they felt challenged the success of these strategies. One area that appeared to be problematic was the law enforcement system. Participants described the issue of laws not being enforced fairly and equally, for example affluent members of society are generally not penalised for violating the law. Similar findings were reported in other studies from India which also highlighted the issue of police accepting bribes for traffic offences [6, 83]. Our study also showed that the violation of traffic laws particularly by law enforcement officials was believed to have a negative influence on the general public.

Aside from a change in the mindset and behaviour of those involved, participants also felt enforcement could be improved through an audit system and by introducing a programme that identifies accident-prone areas so that resources could be channelled appropriately. While India has procedures in place for superintendence and control of the police, there is no system for performance management [84]. Likewise, identification of accident-prone areas while commonly carried out on highways, is not often seen in many cities in India, and it is only until recently that the government has urged all states to identify black spots (a black spot is a stretch of road not more than 500 m in length where five RTCs have taken place or where 10 fatalities have occurred in the last 3 years) as part of the Road Safety Audit [7, 85, 86].

### Limitations

Our study has several limitations with should be noted. The recruitment strategy could have potentially led to selection bias where some of the participants had been research partners or participants of the local team in the past. This could have impacted their response to the questions, given their relationship with the interviewer. Although there was a wide range of participants, it is also possible that other potentially relevant actors from different disciplines, with different views, may have been excluded.

Another limitation was the sample size where only ten participants were included in the final sample. While it is possible to reach saturation with 10 to 15 interviews, where saturation is defined as repeated emergence of a particular theme or themes, which seemed to be the case in our study, it is still possible this number of participants could result in more tentative findings [87].

Most interviews were carried out in the local language and required translation. This made it difficult to carry out member checking for all the transcripts in order to ensure the data was truly representative of what the participants intended to say, as a way of increasing the trustworthiness of the results. Although a local professional service was utilised, transcripts, including those that did not require translation, were in non-standard English with a lot of colloquialisms, which could have affected the reading and interpretation of the data, and posed some difficulties with presenting the data [88].

This study took place in one city in one of the 29 States in India. Each State is diverse geographically, culturally and politically, which could potentially influence perceptions on issues such as RTCs and neurotrauma [89]. As a result, generalisability of our findings may be limited.

### Recommendations

This study highlights the need for qualitative research to be carried out with a wider range of stakeholders, including those from educational institutions and amongst the ‘target populations’, in multiple locations within the country. Further research should also be done to determine objectively the effectiveness of current and proposed interventions in preventing RTCs and neurotrauma.

The results of this study can also be used to develop recommendations for policy, practice and research to improve the prevention of RTCs and neurotrauma in India. These recommendations must be highlighted as they provide an added value to government guidance, namely the 4E’s (Education, Enforcement, Engineering and Emergency Care) strategy proposed by the Indian Ministry of Road Transport and Highways (MORTH) for road safety, and those derived from previous qualitative work [6, 11, 90]. These recommendations can be found in Table 3.

**Table 3:** Recommendations for policy, practice and research

**Education**	
• More regular, consistent educational efforts focusing on groups, populations and areas that are densely populated and prone to RTCs	
• Regular education and training of government officials and entities involved in prevention	
• Introducing road safety education as part of the school syllabus for primary and secondary schools	
• Involving former casualties and their families to spread awareness messages and educate the community	
• Utilising the media in education and awareness	
• Opportunistic education i.e. roadside events and road safety education during vehicle purchase	
• Ensuring education and awareness messages are understood by all layers of society	
**Enforcement and Legislation**	
• Stricter enforcement of laws and penalties for traffic offenses	
• Establishing an audit system to ensure enforcement is carried out consistently and correctly	
• Carrying out targeted enforcement by developing a programme to identify accident-prone areas	
• Eliminating liquor shops near the highways to curb RTCs from drink driving	
**Road Engineering**	
• Ensuring good quality roads are built with adequate width which are multi-lane	
• Establishing a road maintenance system	
• Ensuring speed reduction through road engineering measures such as speed breakers	
**Pre-hospital and Trauma System (Emergency Care)**	
• Organising training programmes for lay first responders which would encourage the public to be involved in providing first aid to RTC casualties	
• Establishing a network for all ambulance services and ensuring staff are adequately trained and ambulances equipped in order to deliver pre-hospital care to casualties	
• Building small first aid centres in villages to assist casualties in rural areas	
• Building more trauma centres which are equipped with neurosurgical expertise in areas where RTCs tend to frequently occur	
• Initiating a system where casualties are transported to the appropriate healthcare centre and not just to the nearest facility	
**Collaborations and Partnerships**	
Developing and maintaining good inter and intra-agency relationships, including non-governmental organisations and the wider community	
**Research**	
• Disseminating research findings through social media or formal reports	
• Scientific conduct of research in more centres by dedicated groups of people	
• Regular audits and reviews of research methods and findings	
• Involving former casualties in research	
• Focusing on the epidemiology of RTCs and secondary neurotrauma prevention	

## Conclusions

RTCs remain a major public health problem in India, and a huge contributor to neurotrauma. This study finds that the main factors that lead to RTCs relate to the behaviour of road users and poor road infrastructure. Although there are various preventative strategies organised by the government and community to tackle these factors, behavioural and implementational deficiencies were highlighted as elements that appear to challenge the success of these programmes. Nevertheless, participants were able to provide recommendations that focused on addressing the gaps and shortfalls in the current approaches, including gaps in research, and reinforcing collective responsibility towards road safety. These actions would be a major step toward improving prevention and reducing the burden of RTCs and neurotrauma in this country.

## Supplementary Information

**Additional file 1.** Standards for Reporting Qualitative Research (SRQR).

**Additional file 2.** Themes, sub-themes and descriptors.

## Data Availability

Data that is freely available is presented in the tables, summaries within the results section, anonymised verbatim quotes from participants and in the additional file. There would be restrictions to accessing original transcripts and any other raw data due to the presence of potentially identifiable information as well as sensitive information. Publicly sharing this data will breach the ethical provisions of this research project. Further approval will be necessary to allow for this data to be made publicly available or re-used in any form, where this approval will need to be sought from the University of Cambridge School of Humanities and Social Sciences (cshsethics@admin.cam.ac.uk).

## Abbreviations

RTCs: Road Traffic Collisions
LMICs: Low and Middle Income Countries
GPONT-RTC: Global Prevention of NeuroTrauma-Road Traffic Collisions
EMS: Emergency Medical Services
