# Supplementary material for: An exploratory qualitative study of the prevention of road traffic collisions and neurotrauma in India: perspectives from key informants in an Indian industrial city (Visakhapatnam)
Source: BMC Public Health. 2021 Mar 30;21:618. doi: 10.1186/s12889-021-10686-z (PMC8008519; doi:10.1186/s12889-021-10686-z)
Supplement: Supplementary file 2 — Additional file 2. Themes, sub-themes and descriptors. [file 12889_2021_10686_MOESM2_ESM.docx]

**ADDITIONAL FILE 2**

**THEMES, SUB-THEMES AND DESCRIPTORS**

| Theme | Sub-theme | Descriptor |
| --- | --- | --- |
| Participants’ knowledge of RTCs in their city, state and/or country | The nature and extent of the problem | - RTCs are a major problem where the occurrence is high amongst adolescents and young adults and tends to involve two-wheelers and pedestrians - RTCs are the leading cause of neurotrauma |
|  | Factors and risk factors leading to RTCs and RTC-related neurotrauma | - Human factors: drink driving, speeding, not using personal safety equipment, wrong-way driving, using mobile devices while driving, under-age driving - Environmental factors: Poor road infrastructure and design, bad weather conditions, being hit by the opposite road user, moving obstacles on the road - Vehicle and equipment factors: Overloaded vehicles, using substandard safety equipment |
| Impact of RTCs | Impact to health and healthcare system | - RTCs can lead to injuries, especially neurotrauma and death - Neurotrauma cannot be cured completely - The increase in RTCs cause an increase in neurotrauma cases which are overwhelming the current healthcare system |
|  | Financial and economic impact | - Loss of family income as RTCs tend to involve the sole breadwinner - National economic impact due to effect on Gross Domestic Product (GDP) and loss of professionals to RTCs |
|  | Social impact | - The city gets a bad name |
| Current preventative strategies | The role of government and related organisations | - Traffic rules and regulations including the Good Samaritan Law - Enforcement of traffic rules by the police - Penalties and fines for and imprisonment of offenders - Educational and awareness programmes about laws and road safety - Training police and lay people to respond to RTCs - Ambulance services with a dedicated signalling system - Trauma centres - Compulsory installation of in-vehicle speed management devices - Speed breakers - Road safety committees |
|  | The role of individuals and communities | - Carrying out educational programmes and awareness campaigns in the community - Collaborating with the government in educational and awareness programmes - Informal educational efforts among friends, family and at the workplace - Building and running a rehabilitation centre |
|  | The role of research | - Research into the severity of RTCs, types of injuries, disabilities and long term impact of RTCs - Some participants are involved in research, for example factors affecting recovery from neurotrauma |
|  | Effectiveness of current strategies | - The government is spending money and doing a lot of work in this area but there are still some deficiencies |
| Challenges to prevention | Physical factors | - Overpopulation or overcrowding resulting in heavy traffic on the roads |
|  | Lack of resources | - Lack of funds - Lack of manpower, especially in law enforcement |
|  | Inconsistent implementation | - Educational and awareness efforts are only carried out in urban areas at educational institutions - Too few trauma centres and concentrated in urban locations - Traffic rules are not being enforced uniformly or equally |
|  | Lack of appropriate action | - Not investigating the root cause of RTCs - RTC victims not transported to the appropriate healthcare facility - Untimely and unsafe road engineering efforts |
|  | Deficiencies in research efforts | - Lack of scientific research - Data not collected in a timely manner - Lack of data sharing - Provision of incorrect information |
|  | Poor governance | - Corruption in the law enforcement system - Lack of political will - Lack of coordination between government agencies - Obstacles from the opposing political parties |
|  | Poor mindset and behaviour | - Disregarding traffic rules - Thinking nothing will happen to them even if rules are violated - Abiding by the rules only to avoid penalties and fines - Forgetting to use personal safety equipment or feeling it is unnecessary for short distances - Irresponsibility amongst parents which affects safety of their children - Misleading advertisements which promote speeding, especially among youth - Law enforcement officials also disobeying traffic rules which influences the behaviour of the community |
|  | Lack of education and awareness | - Unaware of traffic rules and the changes to rules or new rules |

| Perceived recommendations for improving prevention | Enhancing current strategies | - Education and awareness: More targeted education and awareness efforts, commencing road safety education early, using creative and engaging ways to educate and increase awareness, Maximising the use of the media. - Research: More awareness about research among the government entities and the public, widespread dissemination of research findings, investigating the number of victims, causes and on pre-hospital care, utilising former victims and other qualified people in research, auditing research procedures and results - Pre-hospital and trauma system: improving the first responder system with more layperson involvement, first aid centres in rural areas, proper training of ambulance staff and swifter ambulance response time, more decentralised trauma centres with expertise in neurotrauma, appropriate transfer of victims - Enforcement and legislation: Stricter enforcement with an audit system set up and a programme to identify accident-prone areas, small changes in legislation to address current issues, changing the police system - Road engineering: improving road quality and width and ensuring construction is safe, having a system in place for immediate reporting of road defects |
| --- | --- | --- |
|  | Establishing collaborations and partnerships | - There needs to be inter-and intra-agency partnerships and collaborations - There should also be partnership between the government and members of the public |
|  | Changing mindset and behaviour | - Following traffic rules and understanding that it is for safety and not just avoidance of punishment - Citizens should take ownership and responsibility when it comes to preventing RTCs and neurotrauma - The government should have a strong political will and set a good example for citizens |
